# Chronic Pelvic Pain and Infertility Resulting from Unrecognized Retained Laminaria

**DOI:** 10.1155/2017/6345712

**Published:** 2017-08-28

**Authors:** Wesley Nilsson, Sasha Mikhael, Jennifer Kaplan

**Affiliations:** ^1^American University of the Caribbean, Coral Gables, FL, USA; ^2^Department of Obstetrics and Gynecology, Providence Hospital, Michigan State University College of Human Medicine, East Lansing, MI, USA

## Abstract

**Background:**

In 2013, the abortion rate in the United States was found to be 200 abortions per 1,000 live births. Of these, the CDC estimates that nearly 49% were performed using unsafe measures. Even when these procedures are safely performed, patients are at risk for immediate or delayed complications. In second-trimester terminations, mechanical dilation with an osmotic dilator is common to allow for delivery of the fetus. The Japanese seaweed* Laminaria japonica* is used to achieve this purpose.

**Case:**

A 28-year-old primigravida presented with chronic pelvic pain and infertility. She had irregular menstrual cycles and reported scant yellow discharge. A transvaginal ultrasound revealed an abnormally appearing endometrium with an elongated structure suspicious for a foreign body. The patient reported a voluntary termination of pregnancy twelve years earlier, for which laminaria were placed prior to the dilation and extraction. She underwent an operative hysteroscopy confirming our suspicion for retained laminaria. The pathology report demonstrated chronic severe endometritis and plant based material.

**Conclusion:**

Retained laminaria are associated with chronic pelvic pain and chronic infertility. Since they can be difficult to detect on conventional imaging, proper counting prior to insertion and after removal is an essential physician responsibility.

## 1. Introduction

Laminaria are derived from the Japanese seaweed* Laminaria japonica.* Their use in obstetrics began over 100 years ago in the United States [1], and they remain a useful tool today. In the US market, the two types of osmotic dilators available are laminaria and a synthetic version named Dilapan. According to a randomized cohort study done in 1994, there is no advantage to using one dilator over the other [2]. There is, however, evidence that osmotic dilators are effective over other means of cervical ripening such as prostaglandins [3], thus explaining the popularity of their use. Additionally, the Society of Family Planning recommends the preoperative use of cervical dilators to decrease the risk of complications when performing dilation and extraction [4]. Some complications of osmotic dilators such as infection have been reported in the literature, but uncommon amongst the literature is the retention of laminaria. To date, there are no reported cases of retained laminaria leading to infertility.

## 2. Case Report

**Figure 1:**
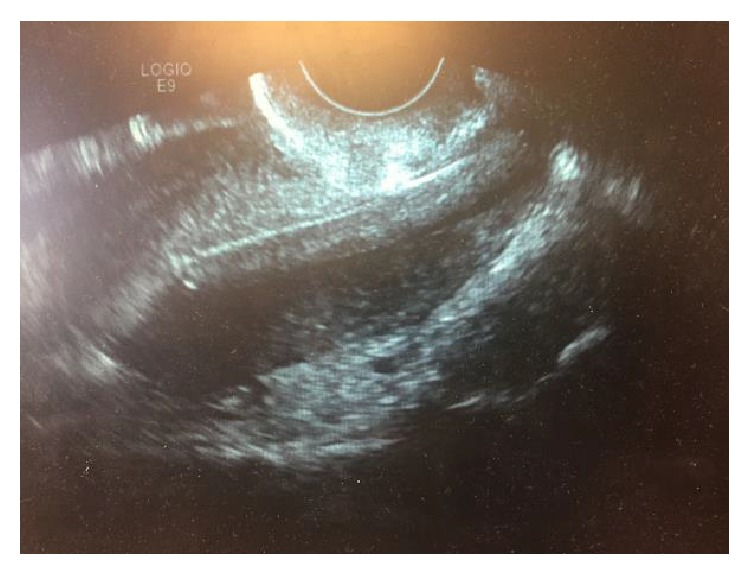
Transvaginal ultrasonography demonstrating retained luminaria.

A 28-year-old primigravid female presented to the emergency department with intermittent pelvic pain for a duration of ten years. The pain recently worsened, prompting an evaluation in the emergency room. In addition to the pain, she disclosed a history of abnormal uterine bleeding and four years of infertility. Sonographic evaluation (Figure 1) demonstrated an abnormally appearing endometrium with an elongated structure within the endometrial cavity. A CT scan confirmed the linear structure embedded within the endometrium but was not useful in narrowing down the differential diagnosis. The patient was discharged home on follow-up as an outpatient.

During her office visit, she reported, as part of her obstetrical history, that a second-trimester termination of pregnancy approximately twelve years ago involved the use of multiple laminaria prior to dilation and extraction. She otherwise denied any additional medical or surgical history. As part of a repeat physical, her pelvic exam revealed yellow, foul-smelling vaginal discharge consistent with a possible foreign body. The patient therefore consented to an operative hysteroscopy with curettage for removal of the suspected foreign body.

**Figure 2:**
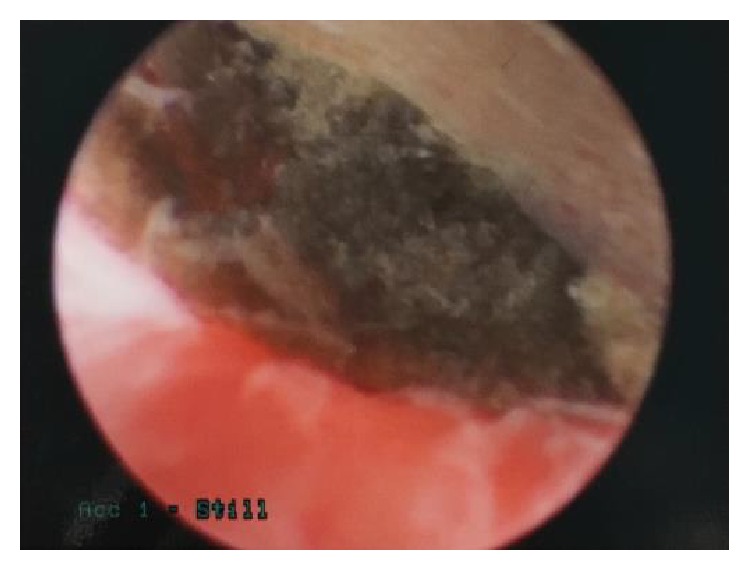
Hysteroscopic view of retained luminaria.

**Figure 3:**
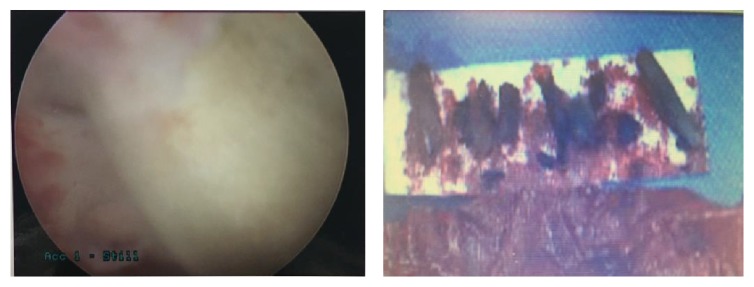
Retained laminaria fragments removed during operative hysteroscopy.

Subsequent hysteroscopic exam revealed extensive endometrial scarring with the embedded foreign body believed to be laminaria (Figure 2). Sharp curettage was performed to break up the adhesions encapsulating the foreign body. With additional use of hysteroscopic graspers, the foreign body was removed in multiple pieces (Figure 3). After successful removal of the laminaria followed by sharp curettage, a Foley catheter balloon was placed within the endometrial cavity to reduce the risk of additional adhesion formation. She was sent home postoperatively with a 30-day regimen of conjugated estrogen in addition to a 10-day course of medroxyprogesterone (to be taken on days 21–30 of the estrogen supplementation).

## 3. Discussion

Osmotic dilators are a well-known and commonly used tool for cervical dilation in the setting of pregnancy terminations. Laminaria are effective, cost-efficient, and relatively safe. Few case reports exist that describe retained laminaria. Of the few that do exist, common patient complaints included pelvic pain and vaginal discharge. Typically, patients reported symptom onset within 24 hours of the procedure [3, 5, 6]. Our case is unique due to the significantly delayed timing of the diagnosis.

Because laminaria can form a dumbbell shape or break or advance into the endometrial canal, there is a risk for retention. If there is retention secondary to these causes, then removal becomes complicated. This is due to the fact that laminaria may be difficult to detect with conventional imaging [7]. Frequently, with retained foreign objects, a significant amount of inflammation and scarring can occur, further obscuring their detection. Borgatta and Barad explained that the presence of blood clots also hindered recognition of laminaria fragments [5]. In the aforementioned scenarios, the use of transvaginal ultrasonography is of little help in confirming the diagnosis. In our case, the retained laminaria were easier to detect; however, due to delayed presentation, radiology workup alone made it difficult to discover the origin of the observed foreign body. A comprehensive history and physical proved to be essential in guiding management with a definitive diagnosis and successful therapy resulting from operative hysteroscopy in conjunction with the pathology report.

## 4. Conclusion

Suggestions have been made regarding imaging techniques for detection of retained laminaria, including the use of sonohysterography as it has been shown to identify laminaria fragments that were not seen using conventional transvaginal ultrasonography. However, a more practicable option includes counting the number of laminaria placed and ensuring they are all intact before placement. Upon removal, it should always be the physician's responsibility to repeat a full count and confirm that all laminaria remain intact. This is ultimately the best technique for the prevention of negative outcomes resulting from retained laminaria. If laminaria retention remains undetected, there is potential for long-term complications such as infertility and chronic pelvic pain, as seen in our patient. Infertility has devastating psychological ramifications, resulting in high levels of anxiety and/or depression [8]. Future workup for this particular patient will involve a follow-up operative hysteroscopy and possibly adhesiolysis for fertility purposes; however, whether she will regain her infertility remains unknown. Even after conception, future obstetrical complications including uterine accreta, increta, or percreta are a cause for concern. To our knowledge, this is the first case of retained laminaria that remained undetected leading to chronic pelvic pain and infertility.
